# Porphyrin Macrocycle Modification: Pyrrole Ring-Contracted or -Expanded Porphyrinoids

**DOI:** 10.3390/molecules21030320

**Published:** 2016-03-09

**Authors:** Letícia D. Costa, Joana I.T. Costa, Augusto C. Tomé

**Affiliations:** Department of Chemistry and QOPNA, University of Aveiro, 3810-193 Aveiro, Portugal; leticia_costa1@msn.com (L.D.C.); jitc@ua.pt (J.I.T.C.)

**Keywords:** porphyrinoids, secochlorins, chlorophins, bacteriophins, azeteoporphyrins, porpholactones, pyriporphyrins, morpholinoporphyrins

## Abstract

In recent years, several synthetic strategies aiming at the peripheral functionalization of porphyrins were developed. Particularly interesting are those involving the modification of β-pyrrolic positions leading to pyrrole-modified porphyrins containing four-, five-, six- or seven-membered heterocycles. Azeteoporphyrins, porpholactones and morpholinoporphyrins are representative examples of such porphyrinoids. These porphyrin derivatives have recently gained an increasing interest due to their potential application in PDT, as multimodal imaging contrast agents, NIR-absorbing dyes, optical sensors for oxygen, cyanide, hypochlorite and pH, and in catalysis.

## 1. Introduction

Porphyrins and porphyrinoid compounds, including contracted and expanded porphyrin derivatives, are highly versatile compounds, both in terms of chemistry (which is extremely rich) and diversity of (potential) applications. These two strands have driven the continuous development of new routes to the synthesis of such compounds.

Porphyrin derivatives are being used in biomedical and environmental applications, in catalysis, and in a range of technical applications [1,2,3,4,5]. Most of these applications require compounds that display absorption bands in the 600–800 nm region. Since “simple” porphyrins hardly fulfill this requirement, chlorins, bacteriochlorins, π-extended porphyrins, pyrrole-modified porphyrins, and other porphyrinoids, are a better choice. Such compounds are typically prepared by: (i) the structural modification of already existing porphyrins via, for instance, cycloaddition reactions, electrophilic or nucleophilic aromatic substitutions, pyrrole ring-contraction or -expansion reactions; or (ii) by constructing the porphyrin macrocycle using adequate pyrrolic building blocks. Considering the last approach, the “3 + 1 method” was extensively used for the synthesis of chlorins and pyrrole-modified porphyrins. This subject has been reviewed recently [6,7,8,9] and will not be covered here. The metallation of porphyrinoids has also been covered in recent reviews [10,11,12].

The modification of the periphery of porphyrins using cycloaddition reactions, namely Diels-Alder reactions and 1,3-dipolar cycloadditions, is a remarkable method to produce chlorins, bacteriochlorins or isobacteriochlorins [13,14,15]. We and other groups have reported several works using porphyrins as dienophiles in Diels–Alder reactions [16,17,18,19,20,21] or as dipolarophiles in 1,3-dipolar cycloadditions [22,23,24,25,26,27,28,29,30,31].

**Figure 1:**
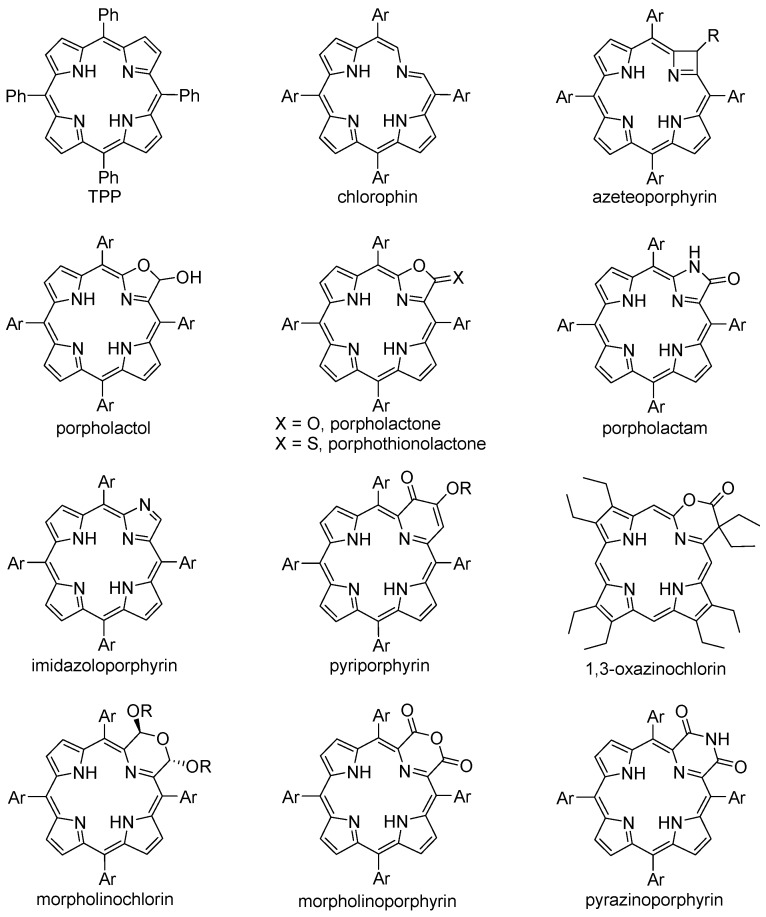
*meso*-Tetraphenylporphyrin (TPP) (for comparison) and examples of pyrrole-modified porphyrins discussed in this review.

Concerning the transformation of porphyrins into pyrrole-modified porphyrinoids, many pyrrole ring-contraction and -expansion reactions were reported during the last three decades. However, a substantial part of that work was published in recent years by the group of Brückner that, by using the “breaking and mending of porphyrins” approach [32], was able to produce an enormous diversity of pyrrole-modified porphyrins (some are exemplified in Figure 1).

This review covers the reported methods for the modification of the porphyrin macrocycle and the potential applications of the resulting porphyrinoid compounds. This subject has already been discussed in recent reviews [33,34].

## 2. Chemistry

The conversion of a porphyrin, or a metalloporphyrin, into a pyrrole-modified porphyrinoid is frequently a multi-step process that requires the separation, purification and structural characterization of the intermediate compounds. However, in this article only the final steps of such transformations are discussed. The methods described below were organized according to the types of porphyrin derivatives used as immediate precursors of pyrrole-modified porphyrinoids.

### 2.1. From N-Substituted Porphyrins

**Scheme 1:**
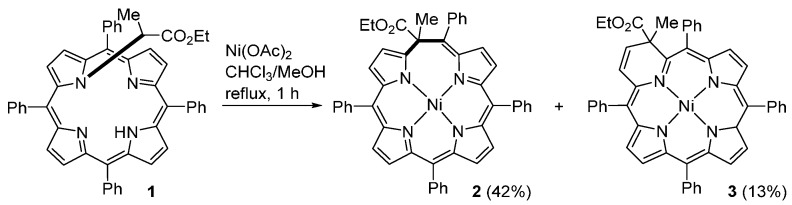
Synthesis of an expanded porphyrin and a pyriporphyrin [35,36].

The metallation of the *N*-substituted porphyrin **1** with nickel acetate in a refluxing CHCl_3_/MeOH mixture leads to the formation of two new porphyrinoids: the expanded porphyrin **2** and the pyriporphyrin **3** (Scheme 1) [35,36]. Compounds **2** and **3** result from the insertion of a C atom in an α–*meso* bond or in an α–β bond, respectively. This reaction, reported by Callott and Schaeffer in 1978, is one of the first examples of the direct conversion of a porphyrin into a pyrrole-modified porphyrin.

### 2.2. From β-Aminoporphyrins

**Scheme 2:**
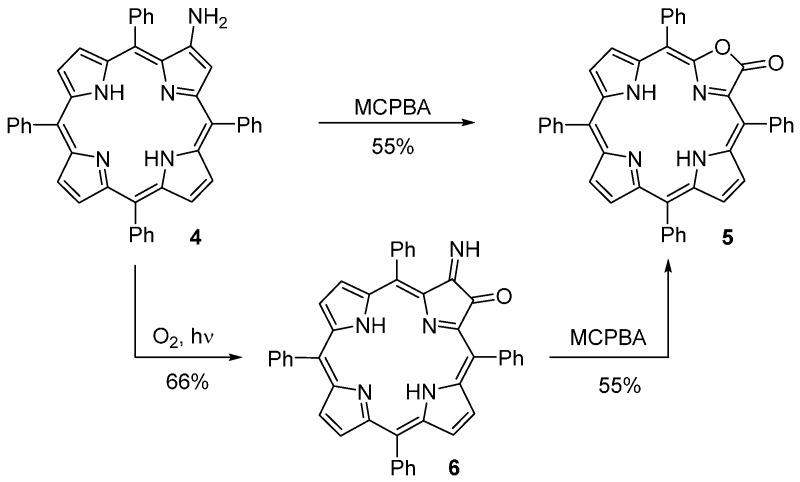
Routes to *meso*-tetraphenylporpholactone reported by Crossley and King [37].

In 1984, Crossley and King [37,38] found that the oxidation of β-amino-*meso*-tetraphenyl-porphyrin (**4**) with *m*-chloroperbenzoic acid (MCPBA) affords porpholactone **5** in 55% yield (Scheme 2). The same porpholactone can be obtained in similar yield by oxidation of the imino-oxochlorin **6** (obtained by photo-oxidation of 2-aminoporphyrin **4**). In their initial communication, these authors also reported the synthesis of a ring-expanded morpholinoporphyrin derivative and a ring-contracted azeteoporphyrin (see Scheme 15).

The method reported by Crossley and King (involving β-nitration of *meso*-tetraarylporphyrins, followed by reduction and oxidation of the resulting β-aminoporphyrins with MCPBA) was used by other groups to prepare porpholactones and the corresponding iron complexes [39].

### 2.3. From 2,3-Dihydroxychlorins

**Scheme 3:**
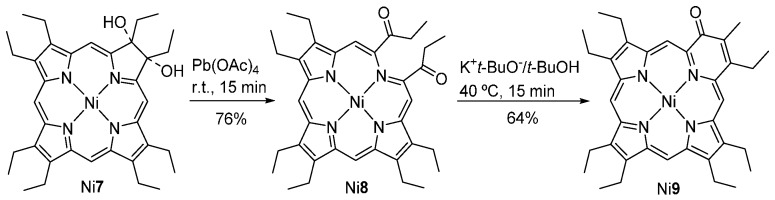
Route to the pyridone-modified porphyrin Ni**9** reported by Bonnett and co-workers [40,41].

In 1993, Bonnett and co-workers [40,41] reported that the oxidative cleavage of the nickel(II) dihydroxychlorin Ni**7** with lead tetraacetate, at room temperature, affords the secochlorin diketone Ni**8** in 76% yield (Scheme 3) [42]. Treatment of Ni**8** with potassium *tert*-butoxide in *tert*-butyl alcohol at 40 °C leads to the formation of the pyridone-modified porphyrin Ni**9** in 64% yield (via an intramolecular aldol condensation). Pb(OAc)_4_ cannot be used in the oxidation of the free-base **7** since it does not allow the isolation of any product in reasonable yield.

**Scheme 4:**
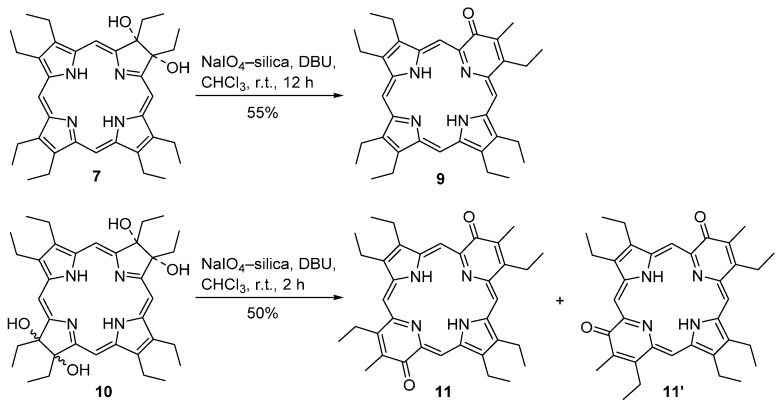
Route to the pyridone-modified porphyrins reported by Brückner and co-workers [43].

Aiming to synthesize free-base pyriporphyrins, Brückner and co-workers used a slurry of silica gel–NaIO_4_ in CHCl_3_ in the presence of 5–10 vol % DBU to convert the free-base dihydroxychlorin **7** to pyriporphyrin **9** (Scheme 4) [43]. This product could be isolated in 55% yield (after chromatographic separation and crystallization) as a purple microcrystalline solid. This method was also applied to the conversion of the tetrahydroxybacteriochlorin **10** into the isomeric bis(pyri)porphyrins **11** and **11’**, which were isolated in a combined yield of 50%.

**Scheme 5:**
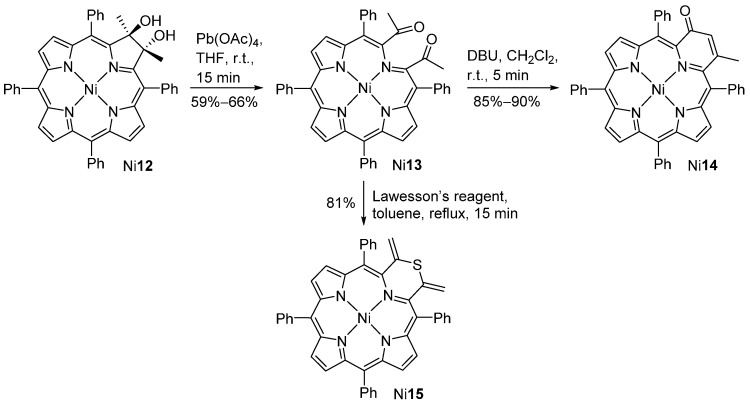
Routes to pyridone- and thiomorpholine-modified porphyrins [44].

Starting from the *trans*-2,3-dimethyl-2,3-dihydroxychlorin Ni**12**, and using lead tetraacetate for the oxidative diol cleavage, Brückner and co-workers were able to generate the 2,3-diacetylsecochlorin Ni**13** (Scheme 5) [44]. Under Brønsted-basic conditions, this diketone cyclizes via an intramolecular aldol condensation to provide the pyriporphyrin derivative Ni**14**. Reaction of the 2,3-diacetylsecochlorin Ni**13** with Lawesson’s reagent induces the formation of the thiomorpholinochlorin Ni**15** substituted with two methylene groups.

**Scheme 6:**
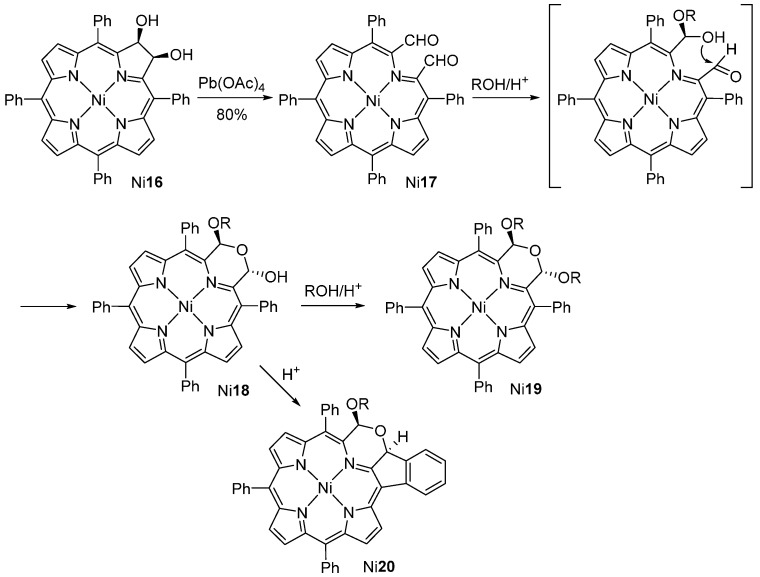
Route to morpholinochlorins [45].

Brückner and co-workers [45,46] also used a 2,3-dihydroxychlorin as an intermediate in the synthesis of porphyrinoids in which one pyrrolic unit is formally replaced by a morpholine ring (Scheme 6). The 2,3-dihydroxy-*meso*-tetraphenylchlorin Ni**16**, obtained by osmium tetroxide-mediated dihydroxylation [47,48] of *meso*-tetraphenylporphyrin (TPP) and complexation with nickel acetate, was transformed into the secochlorin-2,3-dicarbaldehyde Ni**17** by oxidation with lead tetraacetate. The dicarbaldehyde Ni**17** undergoes intramolecular acetal formation when treated with alcohols in the presence of acid to produce a mixture of morpholinochlorins Ni**18** and Ni**19** (see also Scheme 11). Acid treatment of hydroxymorpholinochlorins Ni**18** leads to the establishment of an intramolecular β-to-*o*-phenyl linkage resulting in the formation of the polycyclic-fused porphyrin system Ni**20** [49,50]. Complexes of types Ni**18**, Ni**19** and Ni**20** (and most of their free-bases) exhibit a ruffled macrocycle with an inherent helical chirality. The resolution of the racemic mixtures can be achieved, both by classical methods via diastereomers or by HPLC on a chiral phase [49,50].

A electrochemical study of Ni(II) porphyrinoids showed that, upon electrochemical reduction, morpholinochlorins form ligand-based reduction products while the conformationally flexible chlorin and secochlorin complexes form Ni(I) complexes [51].

**Scheme 7:**
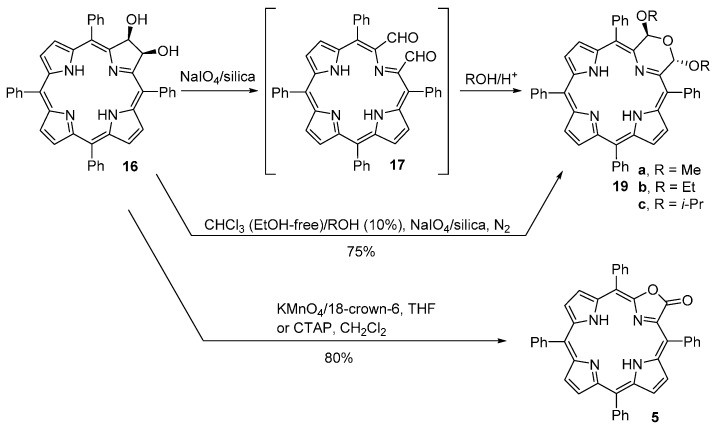
Route to free-base morpholinochlorins and porpholactones [52].

The group of Brückner also reported a variation of the previous method that allowed the synthesis of free-base morpholinochlorins and porpholactones (Scheme 7) [52]. Using the free-base dihydroxychlorin **16** and NaIO_4_ (heterogenized on silica gel) as the oxidant, this group was able to produce, isolate and characterize the unstable secochlorin-2,3-dicarbaldehyde **17**. Reaction of **17** with MeOH, EtOH or *i*-PrOH under acid catalysis provided the stable morpholinochlorins **19a**–**c**. The same compounds can be obtained directly from the reaction of **16** with NaIO_4_/silica under N_2_ in the presence of the corresponding alcohol. Treatment of methoxy derivative **19a** with excess EtOH or *i*-PrOH under acid catalysis at 65 °C leads to alkoxy exchange and formation of **19b** or **19c**, respectively [52]. MnO_4_-induced cleavage of diol **16** under phase transfer catalysis (or using cetyltrimethylammonium permanganate, CTAP) affords porpholactone **5** in overall excellent yield (up to 80%). The formation of **5** probably involves the oxidation of the dicarbaldehyde **17** to the corresponding secochlorin-2,3-dicarboxylate followed by decarboxylation and lactonization [52]. This methodology has been used for the synthesis of a range of porpholactones [53,54]. A similar approach has been used for the conversion of *meso*-tetraphenylporphyrin *N*-oxide into pyrrole-modified porphyrin *N*-oxides [55].

The group of Brückner demonstrated that Ag(II) can serve as a removable metal template in the oxidative cleavage of 2,3-dihydroxychlorins, allowing an easy access to free-base pyrrole-modified porphyrins [56].

**Scheme 8:**
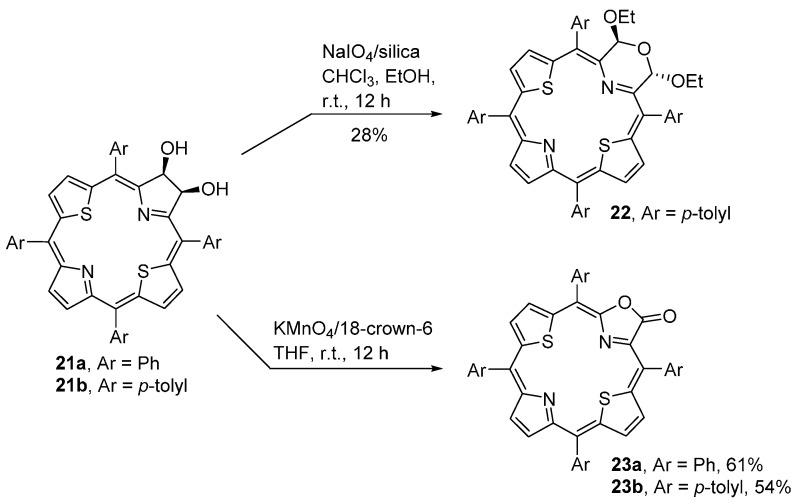
Routes to morpholinodithiachlorins and dithiaporpholactones [58].

The methods described above were also successfully applied to the conversion of dihydroxy-21,23-dithiachlorins **21a**,**b** into the dithiamorpholinochlorin **22** and dithiaporpholactones **23a**,**b** (Scheme 8) [57,58].

**Scheme 9:**
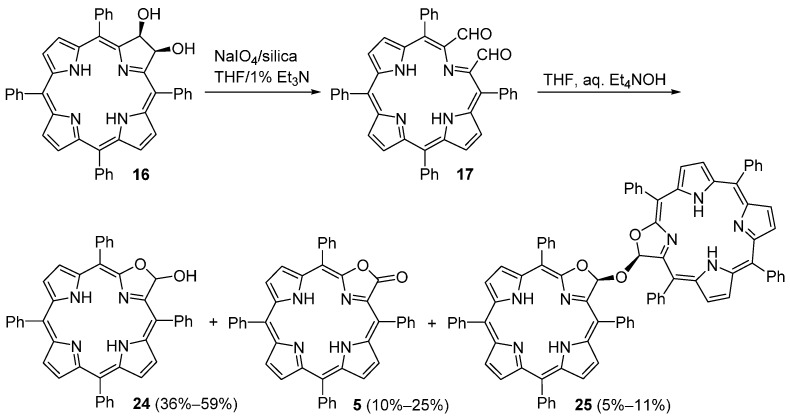
Route to free-base oxazolochlorins [59].

Oxidative cleavage of the purple dihydroxychlorin **16** with NaIO_4_ heterogenized onto silica gel, in THF containing 1–2 vol % Et_3_N, converts it in essentially quantitative yields into the unstable free-base secochlorin-2,3-dicarbaldehyde **17** (a brown nonpolar compound) (Scheme 9) [59]. This dialdehyde can be purified by column chromatography (silica gel, CH_2_Cl_2_–0.1% Et_3_N) but in solution, particularly in acidic and/or wet solvents or on silica gel, it tends to decompose within several hours. However, evaporated to dryness and kept in a freezer at −18 °C, it is stable over several months [59]. When a solution of **17** in THF is treated with a large excess of a 30% aqueous solution of Et_4_NOH, three purple products are formed in varying yields (Scheme 9). The most polar compound (*Rf* = 0.41, silica–CH_2_Cl_2_) is the porpholactol **24**, the one with intermediate polarity (*Rf* = 0.78, silica–CH_2_Cl_2_) is the porpholactone **5** and the least polar one (*Rf* = 0.90, silica–CH_2_Cl_2_) is the porpholactol dimer **25** (isolated in yields up to 11%). The authors proposed a mechanism to rationalize the formation of these products which involves an intramolecular Cannizzaro reaction in the dialdehyde **17** [59].

**Scheme 10:**
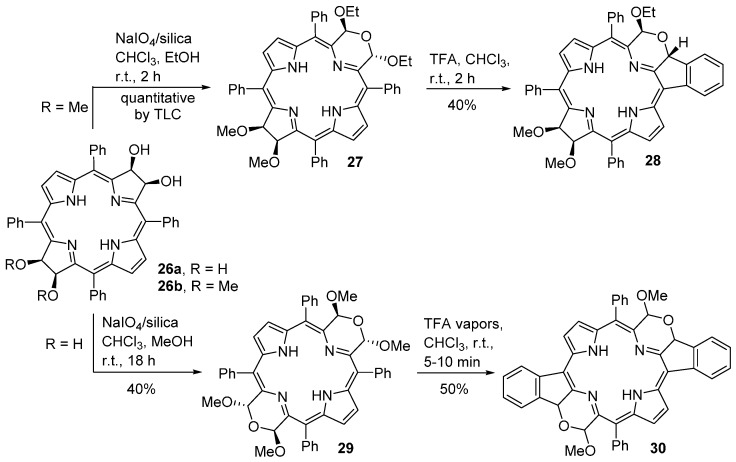
Synthesis of morpholinobacteriochlorins and bis(morpholino)bacteriochlorins [60].

The method described above was also applied to the synthesis of pyrrole-modified bacteriochlorins [60]. Starting from the free-bases 2,3-dihydroxy-12,13-dimethoxychlorin **26b** or the 2,3,12,13-tetrahydroxychlorin **26a** [61], and using mild oxidation conditions (NaIO_4_ heterogenized on silica gel, CHCl_3_, alcohol, room temperature), Brückner and co-workers were able to synthesize the morpholinobacteriochlorin **27** and the bis(morpholino)bacteriochlorin **29**, in one pot, in reasonable yields (Scheme 10). Acid treatment of these morpholinochlorins leads to the formation of the polycyclic-fused porphyrin systems **28** and **30**.

**Scheme 11:**
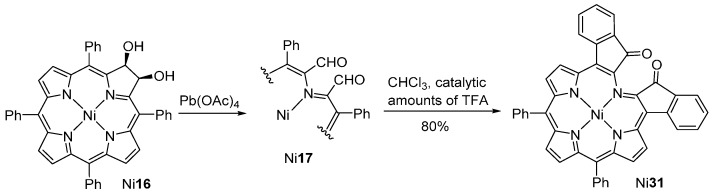
Synthesis of indaphyrins [62].

As shown in Scheme 6, the oxidative cleavage of 2,3-dihydroxy-*meso*-tetraphenylchlorin Ni**16** with lead tetraacetate leads to the formation of morpholinochlorins (via the secochlorin-2,3-dicarbaldehyde Ni**17**). However, depending on the reaction conditions during the ring cleavage reaction, the formyl groups may react with the adjacent *o*-phenyl positions to establish direct *o*-phenyl-to-β-linkages [62,63]. The initially formed carbinols oxidize spontaneously to ketones, resulting in the formation of indaphyrin Ni**31** in high yield (Scheme 11).

**Scheme 12:**
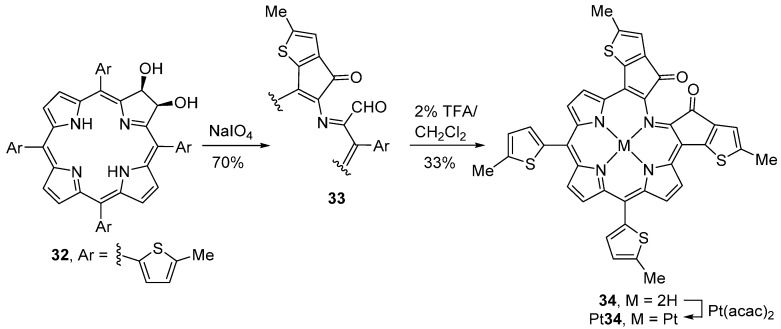
Synthesis of thiaindaphyrins [64].

Free-base dihydroxychlorins can also be used to synthesize indaphyrin-type compounds. In that case, the oxidant should be NaIO_4_ heterogenized on silica gel. As an example, the oxidative cleavage of 2,3-dihydroxy-5,10,15,20-tetrakis(5-methylthien-2-yl)chlorin (**32**) results in the formation of the monothiaindanone monoaldehyde **33** (presumably via a dicarbaldehyde intermediate) (Scheme 12) [64]. Stirring a solution of monoaldehyde **33** in 2% TFA/CH_2_Cl_2_, at room temperature, leads to the formation of thiaindaphyrin **34** in 33% yield. The corresponding platinum(II) complex, Pt**34**, was obtained in 61% yield from the reaction of **34** with [Pt(acac)_2_] (3 equiv.) in PhCN for 5 h at reflux temperature [64].

**Scheme 13:**
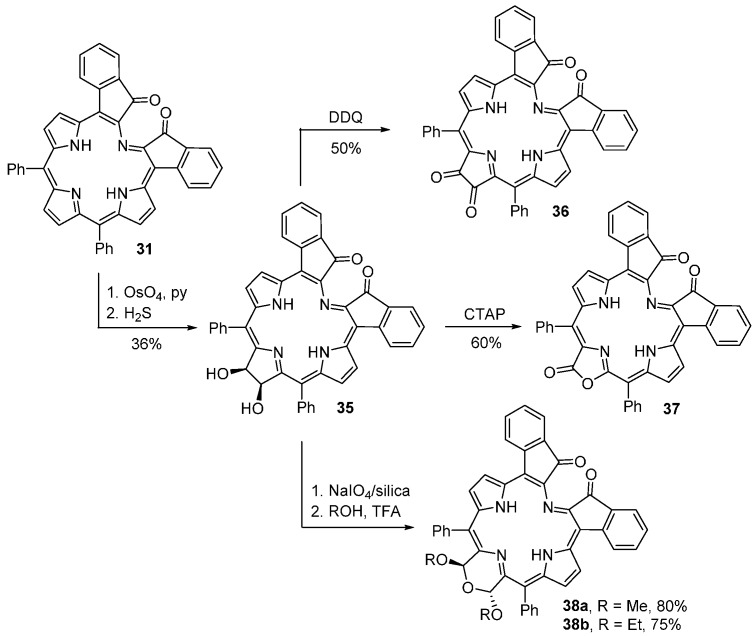
Conversion of an indaphyrin into indachlorins [65].

Indaphyrins can be further functionalized to indachlorins, as indicated in Scheme 13 [65]. Using typical porphyrin dihydroxylation conditions, indaphyrin **31** can be converted into the dihydroxylated indachlorin **35**. Oxidation of **35** with 2,3-dichloro-5,6-dicyano-1,4-benzoquinone (DDQ) leads to the indachlorin dione **36** while oxidation with CTAP affords the corresponding lactone **37**. The dihydroxylated indachlorin **35** can also be converted into dialkoxy-substituted morpholines **38** using standard reaction conditions already described. These compounds display panchromatic absorption spectra between 300 and 900 nm and possess strongly ruffled conformations, incorporating a helimeric twist [65,66]. Resolution of the racemic mixtures of the helimers was achieved by HPLC on a chiral phase and their absolute stereostructures were assigned [66].

**Scheme 14:**
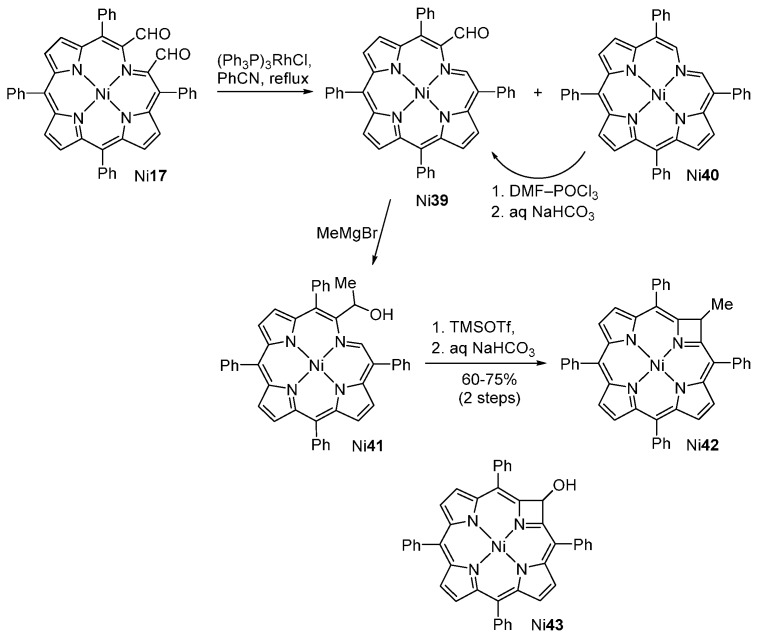
Route to azeteoporphyrins [67].

The Brückner group reported a new approach to pyrrole ring-contracted azeteoporphyrins (Scheme 14) [67]. It involves the oxidative cleavage of the dihydroxychlorin Ni**16** to the secochlorin-2,3-dicarbaldehyde Ni**17** (see Scheme 6) followed by a decarbonylation reaction with an excess of (Ph_3_P)_3_RhCl to afford a mixture of the chlorophins Ni**39** (12% yield) and Ni**40** (60% yield) [46,67]. The monoaldehyde Ni**39** reacts with an excess of methylmagnesium bromide leading to the formation of the secondary alcohol Ni**41**. This alcohol reacts with an excess of TMSOTf to afford the azeteoporphyrin Ni**42** in 60%–75% yield after purification by preparative TLC and crystallization. The role of TMSOTf in this cyclization reaction is to induce the removal of the hydroxyl group and generation of the corresponding carbocation. The azete ring is then formed by an intramolecular Friedel-Crafts reaction.

It is interesting to note that, under adequate experimental conditions, alcohol Ni**41** is obtained in *ca*. 7% yield in the decarbonylation reaction of the dicarbaldehyde Ni**17** [67]. The existence of alcohol Ni**43** had been previously proposed as a side product in the Vilsmeier–Haack formylation of the chlorophin Ni**40** [68].

### 2.4. From 2,3-Dioxochlorins

**Scheme 15:**
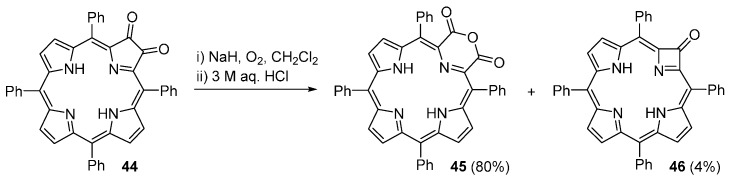
Route to 4- and 6-membered porphyrinoids [37].

In 1984, Crossley and King reported that treatment of a CH_2_Cl_2_ solution of dione **44** (obtained in quantitative yield by acidic hydrolysis of imino-oxochlorin **6**) with an excess of NaH and exposed to air, followed by treatment with 3 M aqueous HCl, affords the morpholinoporphyrin **45** in 80% yield (Scheme 15). The ring-contracted azeteoporphyrin **46** is also formed in this reaction as a minor product [37]. Anhydride **45** can also be obtained in 64% yield by treatment of dione **44** with MCPBA [37].

**Scheme 16:**
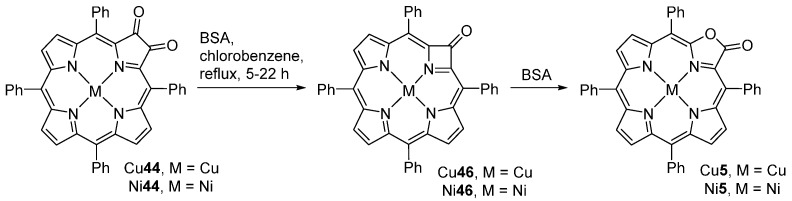
Synthesis of porphyrinoids reported by Zaleski and co-workers [69].

Zaleski and co-workers [69] reported that the Cu(II) and Ni(II) complexes of 2,3-dioxochlorins react with benzeneselenic anhydride (BSA) leading to the formation of ring-contracted azetine derivatives M**46** that further react with BSA to afford porpholactones M**5** (Scheme 16). The yields of compounds M**46** and M**5** are highly dependent on the 2,3-dioxochlorin/BSA ratio and reaction time. When the reaction is carried out in a 1:2 ratio of M**44** to BSA, the reaction proceeds very slowly and after 22 h both the ring-contracted azetine and the porpholactone are obtained in low yields while 59% of the unreacted dioxochlorin is recovered (Table 1). However, if a large excess of BSA is used (8-fold) all starting material is consumed within 5 h. The experimental results show that the isolated yield of the ring-contracted product is consistently low, suggesting that this species is an intermediate to the porpholactone M**5**. In fact, addition of 4 equivalents of BSA to a refluxing chlorobenzene solution of Cu**46** generates Cu**5** within 30 min; the reaction is complete after 16 h, resulting in 59% yield of Cu**5**.

**Scheme 17:**
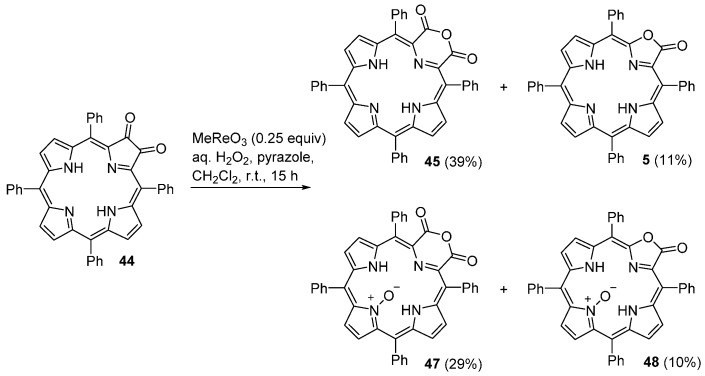
Synthesis of porphyrinoids and the corresponding *N*-oxides [55].

The methyltrioxorhenium (MTO)-catalyzed H_2_O_2_ oxidation of the 2,3-dioxochlorin **44**, in the presence of pyrazole, leads to the formation of four pyrrole-modified porphyrin derivatives (Scheme 17) [55,70]. During the reaction course it is observed the initial formation of compounds **45** and **5** while the corresponding *N*-oxides **47** and **48** are formed later in the reaction, and on the expense of the products formed initially [55]. The yield of each product depends on the reaction conditions, namely catalyst loading and reaction time.

**Scheme 18:**
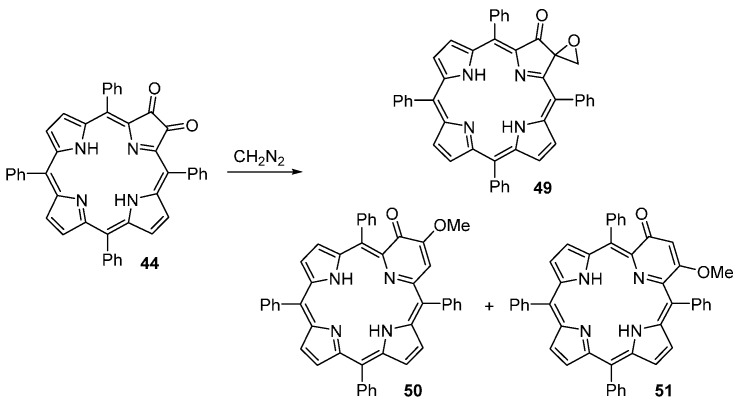
Synthesis of 2-oxopyriporphyrins [71].

**Scheme 19:**
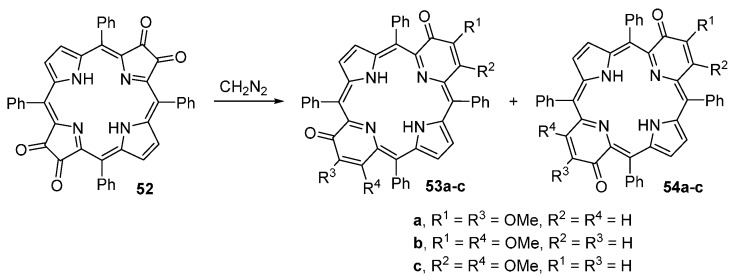
Synthesis of di(2-oxopyri)porphyrins [71].

Pandey and co-workers reported a versatile approach to mono- and di(2-oxopyri)porphyrins (Scheme 18 and Scheme 19) [71]. The new compounds are obtained from the reaction of dioxo- and tetraoxo-TPP derivatives with a large excess of diazomethane. The reaction of dioxochlorin **44** with diazomethane affords three products that can be separated by chromatography on silica gel: the 2-oxo-3-epoxymethylenechlorin **49** (7% yield), 3-methoxy-2-oxopyriporphyrin **50** (12% yield) and 4-methoxy-2-oxopyriporphyrin **51** (78% yield). The tetraoxobacteriochlorin **52** reacts immediately with diazomethane to give a mixture of three orange-brown bands that were identified as a mixture of two isomeric di(oxopyri)porphyrins: **53a**/**54a** (18% yield), **53b**/**54b** (31% yield), and **53c**/**54c** (47% yield) (Scheme 19).

**Scheme 20:**
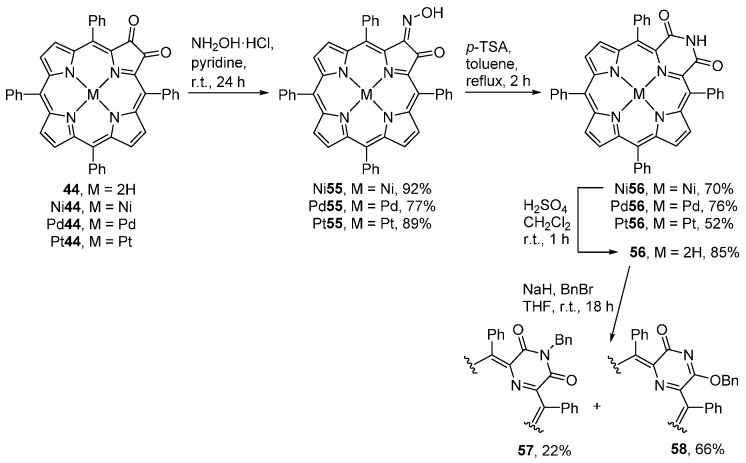
Synthesis of oximes, Beckmann rearrangement to pyrazinoporphyrin imides and benzylation [72].

The reaction of 2,3-dioxoporphyrin metal complexes M**44** (obtained by metallation of **44**) with hydroxylamine hydrochloride affords the corresponding monooximes M**55** in good yields (Scheme 20) [72]. When treated with *p*-toluenesulfonic acid (*p*-TSA) under forcing conditions, oximes M**55** undergo a Beckmann rearrangement to produce the corresponding pyrazinoporphyrin imides M**56** in moderate to good yields. Demetallation of pyrazinoporphyrin Ni**56** affords the free-base pyrazinoporphyrin imide **56**. The reaction of **56** with benzyl bromide in the presence of sodium hydride under nitrogen, at ambient conditions, leads to a mixture of the *N*-benzyl (22%) and *O*-benzyl (66%) derivatives **57** and **58**, respectively.

Treatment of the free-base oxime **55** (M = 2H) with *p*-TSA in toluene at reflux leads to the formation of quinoline-annulated porphyrins [73,74].

### 2.5. From 2-Diazo-3-Oxochlorins

2-Diazo-3-oxochlorins (that can be prepared in good yields from 2-aminoporphyrins [75] or 2,3-dioxochlorins [76]) undergo photodecomposition, with extrusion of N_2_, to yield a mixture of porphyrin derivatives. The product distribution strongly depends upon the central metal ion and the presence or absence of nucleophilic substrates [77,78,79,80].

**Scheme 21:**
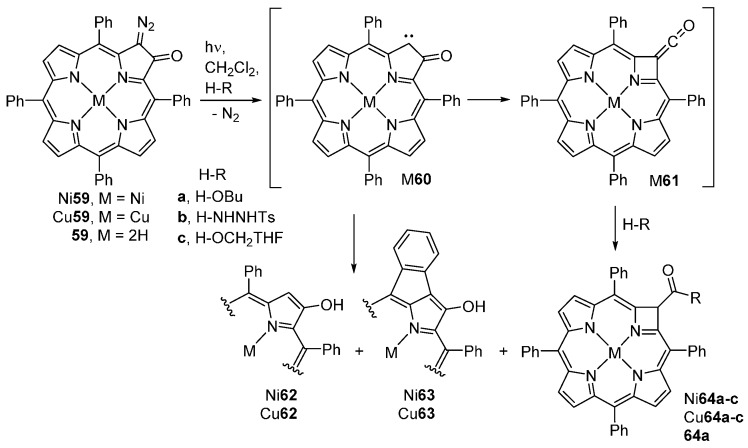
Products generated by photolysis of 2-diazo-3-oxo-5,10,15,20-tetraphenylchlorins [79].

The photolysis of metallated 2-diazo-3-oxochlorins M**59** in the absence of nucleophiles affords a mixture of 2-hydroxyporphyrins (M**62**) and exocyclic ring-containing hydroxyporphyrins (M**63**) (Scheme 21). In the presence of nucleophiles, the ring-contracted azeteoporphyrins M**64** are also obtained. For instance, photolysis of Ni**59** in the presence of butan-1-ol, tosylhydrazide, or tetrahydrofurfuryl alcohol yields the Wolff rearranged azeteoporphyrins **Ni64a**–**c** (11%–28%), the 2-hydroxyporphyrins Ni**62a**–**c** (6%–35%), and the intramolecular exocyclic ring-containing hydroxyporphyrinoids Ni**63a**–**c** (12%–76%) [79]. The photolysis of the free-base 2-diazo-3-oxochlorin **59** in the presence of BuOH affords azeteoporphyrin **64a** (34%) and a dimeric porphyrin derivative (10%). The formation of ring-contracted azeteoporphyrins M**64** involves the Wolff rearrangement of ketocarbene M**60** to the ketene M**61** that is subsequently trapped with the nucleophiles [81].

### 2.6. From Octaethyl-2-oxochlorins

**Scheme 22:**
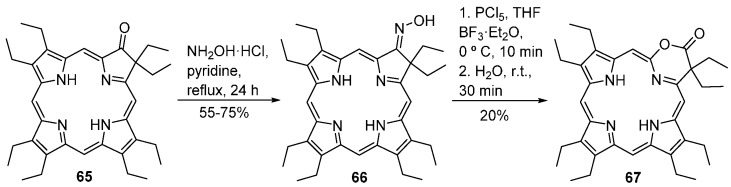
Route to 1,3-oxazinochlorins [82].

The synthesis of the first 1,3-oxazinochlorin (a pyrrole-modified porphyrin with an 1,3-oxazine ring) was reported recently [82]. The synthetic route involved the conversion of the oxo-chlorin **65**, available from octaethylporphyrin, into the corresponding oxime **66** followed by treatment with PCl_5_ and BF_3_·Et_2_O and dropwise addition of H_2_O under controlled temperature (<25 °C) (Scheme 22). The resulting 1,3-oxazinochlorin **67** was obtained in 20% yield together with some ketone **65** (~20%).

### 2.7. From 2,3,12,13-Tetrabromoporphyrins

**Scheme 23:**
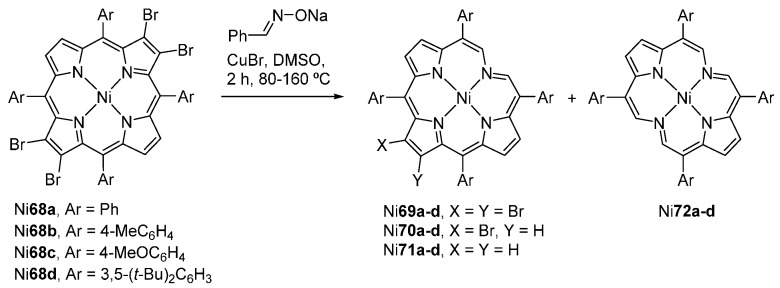
Route to chlorophins and bacteriophins [83].

Nickel(II) 2,3,12,13-tetrabromo-5,10,15,20-tetraarylporphyrins Ni**68a**–**d** react with the anion of *E*-benzaldoxime, in the presence of CuBr, to provide the corresponding chlorophins Ni**69**–Ni**71** and bacteriophins Ni**72**, resulting from the degradation of one or two pyrrolic units, respectively, and mono- or didebromination (Scheme 23) [42,83]. The yield of each product is highly dependent on the time and temperature of the reaction: the best yield of Ni**72a** (26%) is achieved after 2 h at a temperature fluctuating regularly between 80 °C and 160 °C. These chlorophins and bacteriophins display UV-vis spectra similar to those of metallated chlorin and bacteriochlorin systems but with more intense and bathochromically shifted Q-bands.

### 2.8. From β-Unsubstituted Porphyrins

**Scheme 24:**
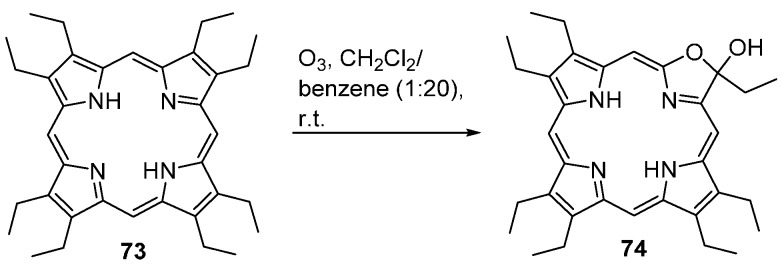
Route to oxazolochlorins [84].

Octaethylporphyrin (**73**) reacts with ozone at room temperature to afford the oxazolochlorin **74** (Scheme 24) [84]. This is probably the first reported example of a direct conversion of a porphyrin into a pyrrole-modified porphyrin.

**Scheme 25:**
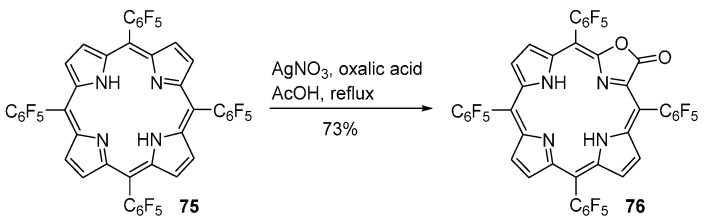
Route to *meso*-tetrakis(pentafluorophenyl)porpholactone [85,86].

Gouterman and co-workers discovered, by serendipity, the direct conversion of free-base *meso*-tetraarylporphyrins into porpholactones using mild reagents [85]. While attempting to synthesize the silver complex of *meso*-tetrakis(pentafluorophenyl)porphyrin (AgF_20_TPP) by treatment of the corresponding free-base H_2_F_20_TPP **75** with AgNO_3_ in glacial acetic acid at reflux, they obtained (in low yield) the corresponding porpholactone **76** (Scheme 25). These authors found that addition of oxalic acid to the reaction mixture allows the synthesis of porpholactone **76** in reproducible yields (*ca*. 15%) [85]. Later, the yield of this reaction was improved to 73% [86]. Porpholactone **79** can be converted into the corresponding Ni, Zn, Pd and Pt complexes by standard procedures [86].

**Scheme 26:**
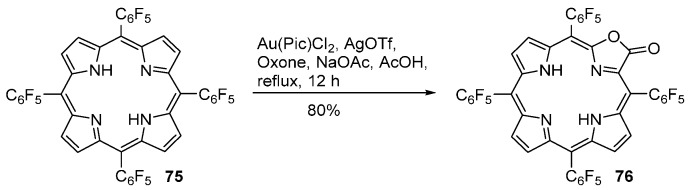
Gold-catalyzed synthesis of *meso*-tetrakis(pentafluorophenyl)porpholactone [87].

Zhang and co-workers [87] found that heating a solution of H_2_F_20_TPP **75** and HAuCl_4_ (2 equiv.) in acetic acid at reflux affords a mixture of [Au(F_20_TPP)]^+^ (20% yield), 2-chloro-H_2_F_20_TPP (25% yield) and the porpholactone **76** (6% yield). However, under similar conditions, TPP affords only the corresponding gold complex [AuTPP]^+^ in 85% yield. Using 2-picolinic acid (Pic) as ligand, the porpholactone **76** is obtained in 63% yield (yield calculated based on the conversion (52%) of the starting porphyrin). A further improvement of the porpholactone yield is achieved by adding an oxidant to the reaction mixture. The highest yield of porpholactone **76** (80%) is obtained when two equivalents of Oxone^®^ are used, although the conversion of H_2_F_20_TPP was low (64%) (Scheme 26). Under these conditions, the complex [AuF_20_TPP]^+^ is formed in very low yield (<2%). In the absence of [Au(Pic)Cl_2_], the reactivity of Oxone^®^ toward H_2_F_20_TPP is very low (<5%), confirming the catalytic effect of Au(III). Other fluorinated *meso*-tetraarylporphyrins can be converted into porpholactones by this gold-catalyzed approach. However, the electronic effect of the substituents of the porphyrins is very pronounced since the yield of the gold porphyrin complex increases and consequently the yield of the porpholactone reduces drastically in the order: *meso*-tetrakis(tetrafluorophenyl)-, *meso*-tetrakis(trifluorophenyl)-, *meso*-tetrakis(difluorophenyl)-, and *meso*-tetrakis(*p*-fluorophenyl)porphyrin. The electronic effect of the substituents may be minimized if Ag^+^ is used as a template metal ion. Starting from AgTPP, Ag(*p*-ClTPP) and Ag(*p*-FTPP) the corresponding free-base porpholactones are obtained in 23%, 17% and 10% isolated yields [87].

**Scheme 27:**
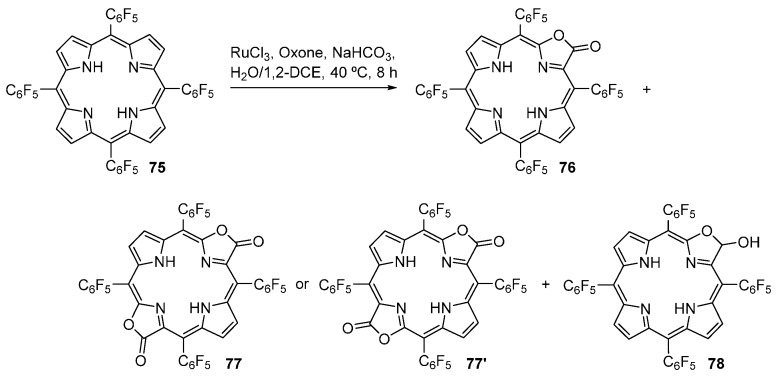
Ruthenium-catalyzed oxidation of *meso*-tetrakis(pentafluorophenyl)porphyrin [88,89].

Zhang and co-workers also investigated the efficacy of RuCl_3_ as catalyst in the direct conversion of *meso*-tetraarylporphyrins to the corresponding porpholactones [88,89]. Using H_2_F_20_TPP as substrate, RuCl_3_ as catalyst and Oxone^®^/NaHCO_3_ as oxidant, these authors obtained a mixture of porpholactone **79** (32% yield), porphodilactones **77**/**77’** (4% yield) and porpholactol **78** (2% yield) (Scheme 27). This reaction system was optimized in order to improve the reactivity and chemoselectivity. The best yield of porpholactone **76** (85%) was obtained using RuCl_3_ (20 mol%), bipyridine (20 mol %, as a ligand), Oxone^®^ (5 equiv.) and sodium hydroxide (5 equiv.) and a reaction temperature of 60 °C.

**Scheme 28:**
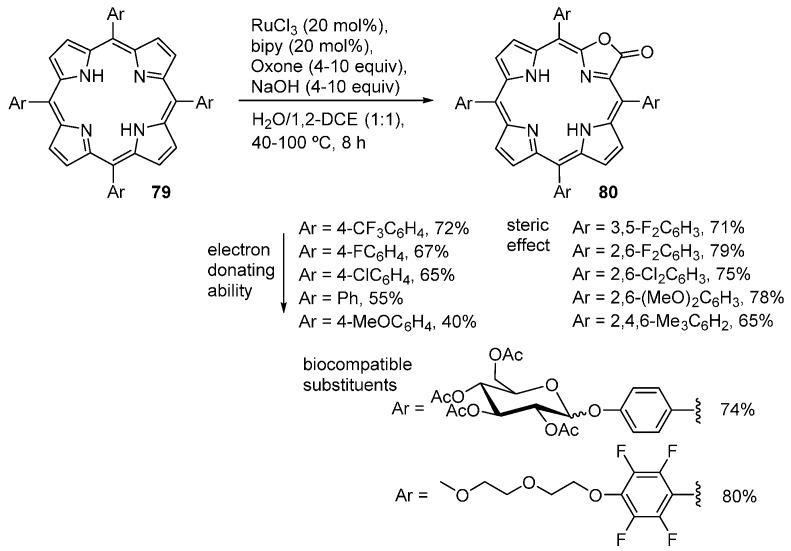
Ruthenium-catalyzed oxidation of *meso*-tetraarylporphyrins [88].

This methodology can also be applied successfully to *meso*-tetraarylporphyrins bearing substituents with different electronic and steric effects and biocompatible substituents, as shown in Scheme 28. The corresponding porpholactones **80** are obtained in 40%–80% isolated yields [88]. Metalloporphyrins can also be converted into metalloporpholactones by this protocol. Using MF_20_TPP (M = Ni, Cu, Zn, Pd) as substrates, the corresponding metalloporpholactones are obtained in high yields (78%–85%). However, oxidation of PtF_20_TPP gives a mixture of Pt(porpholactone) (30% yield) and the free-base porpholactone (46% yield) while no gold(III) porpholactone is detected from the oxidation of [AuF_20_TPP]Cl [88].

### 2.9. Modification of Other Porphyrinoids

**Scheme 29:**
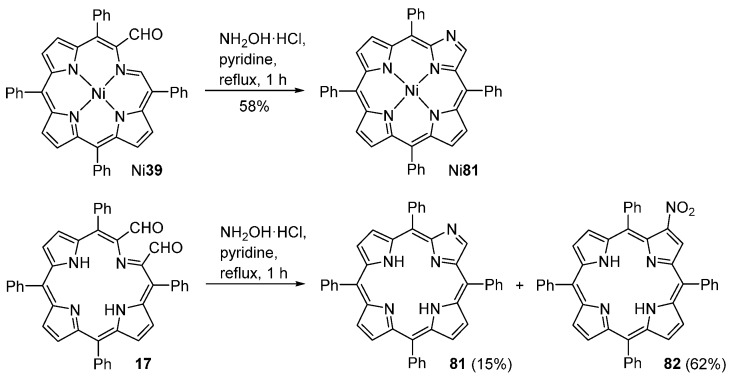
Conversion of chlorophins and secochlorins into imidazoloporphyrins [90].

The reaction of chlorophin-2-carbaldehyde Ni**39** with an excess of hydroxylamine hydrochloride in refluxing pyridine, for 1 h, generates the imidazoloporphyrin Ni**81** in 58% yield (Scheme 29) [90]. The free-base imidazoloporphyrin **81** was obtained in 15% yield from the reaction of secochlorin-2,3-dicarbaldehyde **17** with hydroxylamine hydrochloride in refluxing pyridine. The main product of this reaction is, however, 2-NO_2_TPP (**82**, 62%).

**Scheme 30:**
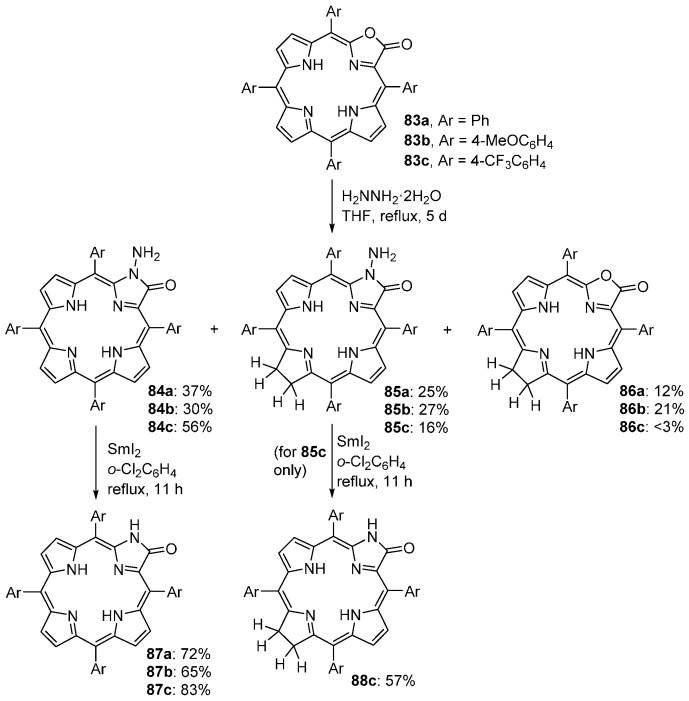
Conversion of porpholactones into *N*-aminoporpholactams and porpholactams [92].

*meso*-Tetraarylporpholactones **83** react with hydrazine hydrate, in THF solution, to form three products: the *N*-aminoporpholactams **84** (main products), the *N*-aminochlorolactams **85**, and the chlorolactones **86** (minor products) (Scheme 30) [91,92]. The reaction requires a large excess of hydrazine hydrate and heating at reflux for 3–5 days. The reductive cleavage of the N-N bond in *N*-aminoporpholactams **84** or *N*-aminochlorolactams **85**, using SmI_2_ in refluxing *o*-dichlorobenzene, affords the parent porpholactams **87** or **88**, respectively, in good yields. 

**Scheme 31:**
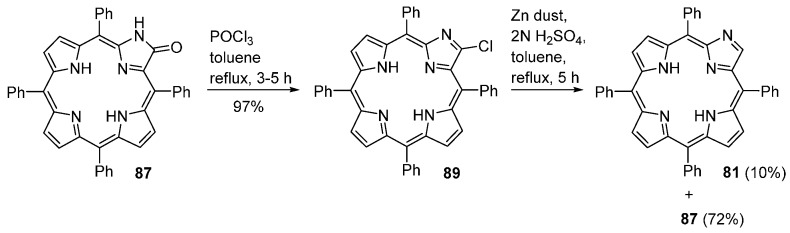
Conversion of porpholactams into imidazoloporphyrins [92].

Porpholactams can be further modified to imidazoloporphyrins, as shown in Scheme 31. In fact, treatment of porpholactam **87** with phosphoryl chloride (POCl_3_) in refluxing toluene gives access to the 3-chloroimidazoloporphyrin **89** in 97% yield. Subsequent reaction of a toluene solution of **89** with zinc dust and 2 N aq. H_2_SO_4_, at reflux for 5 h, affords a mixture of imidazoloporphyrin **81** (10% yield) and the starting porpholactam **87** (72%) [92].

**Scheme 32:**
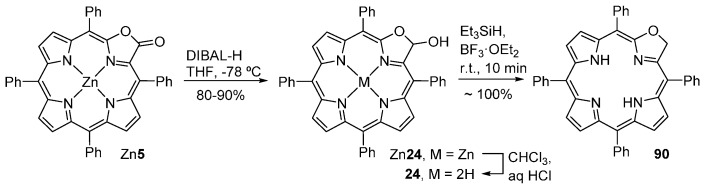
Stepwise reduction of a porpholactone to a porpholactol and a 2-oxachlorin [94].

Reduction of the porpholactone Zn**5** with DIBAL-H affords the porpholactol Zn**24** that, by demetallation, provides the free-base porpholactol **24** in almost quantitative yield (Scheme 32) [54,93,94]. The acid-catalyzed (BF_3_·OEt_2_ or Amberlyst 15) deoxygenation of the porpholactol **24** affords the 2-oxachlorin **90** in near-quantitative yield. Over time, this compound undergoes (photo)oxidation back to porpholactol **24**. Thus, it must be shielded from exposure to light or oxidizing conditions. Attempts at one-step reductions from Zn**5** to Zn**90** using LiAlH_4_ or DIBAL-H under more forcing conditions failed, leading to the destruction of the porphyrinic macrocycle [54].

**Scheme 33:**
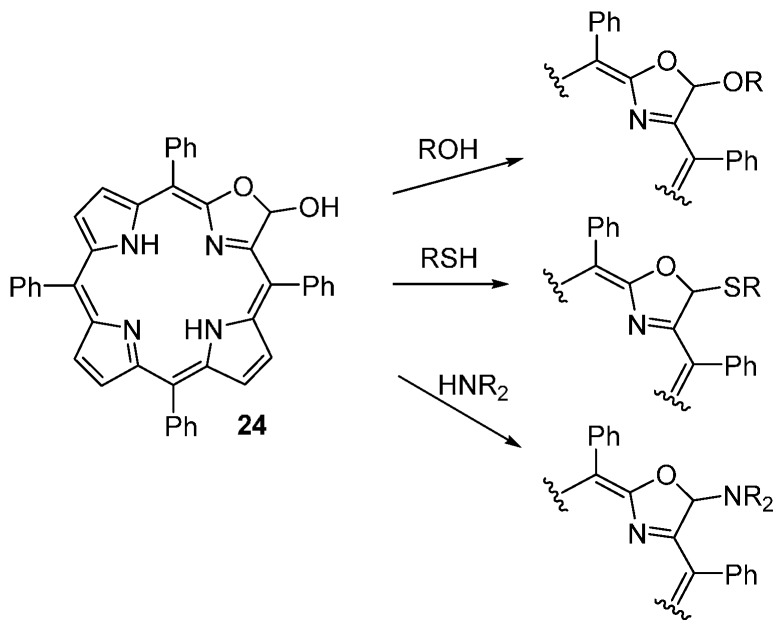
Derivatization of porpholactol **24** with *O*-, *N*-, and *S*- nucleophiles [54].

The porpholactol **24** (an hemiacetal) reacts with a range of *O*-, *N*-, and *S*- nucleophiles, under mild acid-catalyzed conditions, providing access to a number of stable chlorin-like acetals, thioacetals, and aminals (Scheme 33) [54]. 

**Scheme 34:**
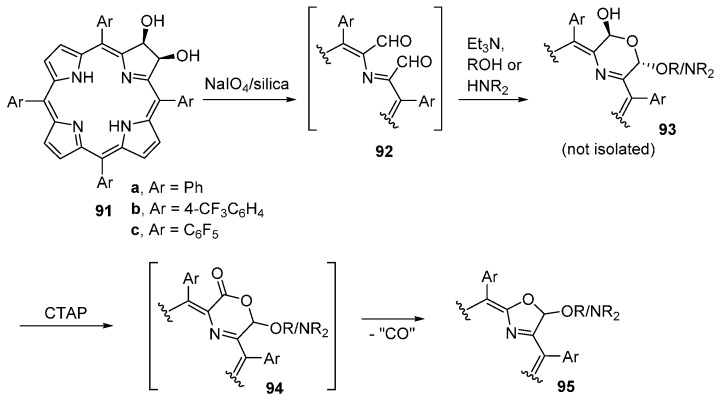
One-pot synthesis of oxazolochlorin acetals and aminals [95].

Acetals and aminals **95** can be prepared in a two-step, one-pot reaction directly from 2,3-dihydroxychlorins **91** (Scheme 34) [95]. The first step corresponds to the oxidation of 2,3-dihydroxychlorins **91** to secochlorin-2,3-dicarbaldehydes **92** (see Scheme 7) in the presence of a nucleophile (alcohol or secondary amine). The resulting crude mixture of morpholinochlorins **93** is then oxidized with CTAP. Depending on the nucleophile used in the first step, oxazolochlorin acetals or aminals are formed in good to acceptable isolated yields. The mechanism of the conversion of morpholinochlorins **93** into the oxazolochlorin derivatives **95** probably involves the formation of morpholinones **94** and extrusion of CO. Comparing this synthetic route to acetals and aminals with the previous one, the number of steps was reduced from 5 to 2 and the overall yields more than doubled: 73%–91% for the acetals and 33%–49% for the aminals.

**Scheme 35:**
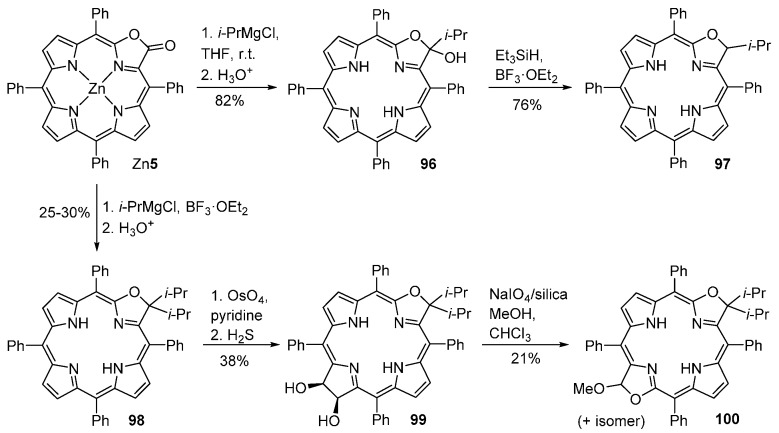
Addition of alkyl-Grignard reagents to porpholactones and further transformation into bisoxazolobacteriochlorins [96,97,98].

Porpholactones react with alkyl-Grignard reagents to afford 3-alkyl- or 3,3-dialkyloxazolochlorins [96,97,98]. Addition of *i*-PrMgCl (15 equivalents) to the zinc complex of porpholactone Zn**5**, followed by an acid workup (that also removes the Zn(II)), affords the hemiketal **96** in 82% yield (Scheme 35). Reaction of **96** with an excess of Et_3_SiH, in the presence of BF_3_·OEt_2_, leads to the formation of the 3-isopropyloxazolochlorin **97** in 76% yield. Addition of an excess of *i*-PrMgCl to porpholactone Zn**5** in the presence of BF_3_·OEt_2_ gives the diisopropyloxazolochlorin **98** in *ca*. 30% yield. Oxazolochlorins **96**–**98** can be further converted into bacteriochlorin-type compounds, such as **99** and **100** [96,97,98]. Oxazolochlorins **96**–**98** and the corresponding hydroxylated oxazolobacteriochlorins possess typical chlorin and bacteriochlorin-like optical spectra with absorption bands between 650–750 nm.

**Scheme 36:**
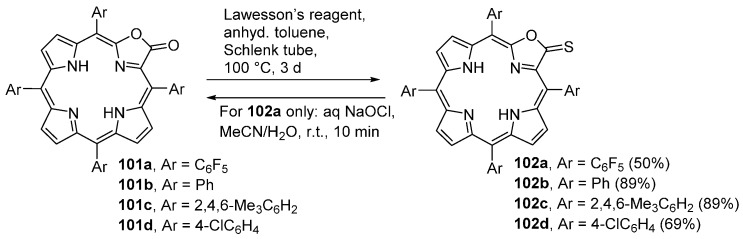
Conversion of porpholactones into porphothionolactones [99].

Reaction of porpholactones **101a**–**d** with an excess of Lawesson’s reagent leads to the formation of the corresponding porphothionolactones **102a**–**d** in good to excellent yields (Scheme 36) [99]. The porphothionolactone **102a** can be converted to the corresponding lactone **101a** rapidly and quantitatively by addition of aqueous NaOCl (bleach). Since porpholactones are moderately fluorescent and the porphothionolactones are not, any reaction that induces the thionolactone-to-lactone conversion is accompanied by a strong fluorescence emission intensity enhancement. This process is the basis for the use of thionolactone **102a** as a switch-on chemodosimeter for hypochlorite (see Section 3.4. Optical Sensors).

**Scheme 37:**
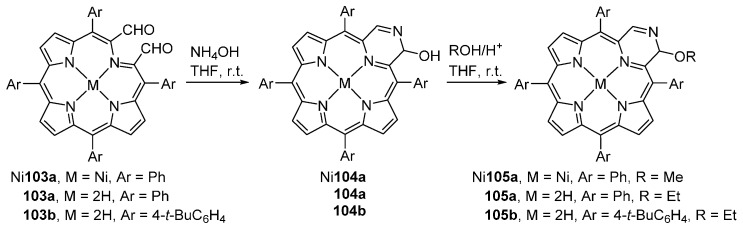
Route to pyrazinoporphyrins [100].

Reaction of secochlorin Ni**103a** with aqueous concentrated ammonia in THF, at room temperature, for 20 minutes, generates the pyrazinoporphyrin hemiacetal Ni**104a** in ca. 90% yield. Treatment of crude Ni**104a** with MeOH and catalytic quantities of THF generates the methoxy pyrazinoporphyrin Ni**105a** (Scheme 37) [100]. The free-base pyrazinoporphyrins **105a**,**b** can be prepared in a similar way. Treatment of the secochlorins **103a**,**b** under aerobic conditions with an excess of aqueous concentrated ammonia in pyridine at 40–50 °C, leads to the rapid formation of the pyrazinoporphyrin hemiacetals **104a**,**b**. While the Ni(II) complex Ni**105a** is stable even under acidic conditions, the free-base analogues are very sensitive to decomposition, particularly under acidic or oxidative conditions. The extinction coefficients of the pyrazinoporphyrins are significantly smaller than those of TPP.

## 3. Applications

Pyrrole-modified porphyrin derivatives have recently gained an increasing interest due to their potential application in photodynamic therapy (PDT), NIR-absorbing dyes, multimodal imaging contrast agents, catalysis, high pH sensors, optical sensors for cyanide, hypochlorite, and oxygen.

### 3.1. Photodynamic Therapy

Photodynamic therapy (PDT) is a promising strategy in cancer treatment. It uses a photosensitizer that, upon cellular internalization and light irradiation, generates reactive oxygen species (ROS) (mainly singlet oxygen) that kill the cancer cells leading to the eradication of the tumor. Porphyrin derivatives, namely chlorins and bacteriochlorins, due to their photophysical properties, such as strong absorption bands in the range of 650–800 nm, and appropriate triplet state to generate ROS, are particularly useful as photosensitizers. Pyrrole-modified porphyrins that display absorption spectra typical of chlorins or bacteriochlorins are also interesting compounds to be used as photosensitizers in PDT. Recent studies confirm the superior properties of such compounds.

In 2005, McCarthy and co-workers described the encapsulation of *meso*‑tetraphenylporpholactol (**24**), a chlorin-type compound, into a poly(lactic-*co*-glycolic acid) (PLGA) matrix and the use of the resulting nanoparticles as photosensitizers in the PDT of tumors [94]. These authors found that the PLGA/porpholactol nanoparticles are unable to fluoresce or induce phototoxicity. However, upon cell internalization, the porpholactol is released from the nanoparticle, regains its ability to fluoresce and produce singlet oxygen, and becomes highly phototoxic. Irradiation with visible light results in cell-specific killing of several cancer cell lines. Importantly, in vivo experiments with this activatable PDT-nanoagent resulted in the complete eradication of cancers in mouse models.

**Scheme 38:**
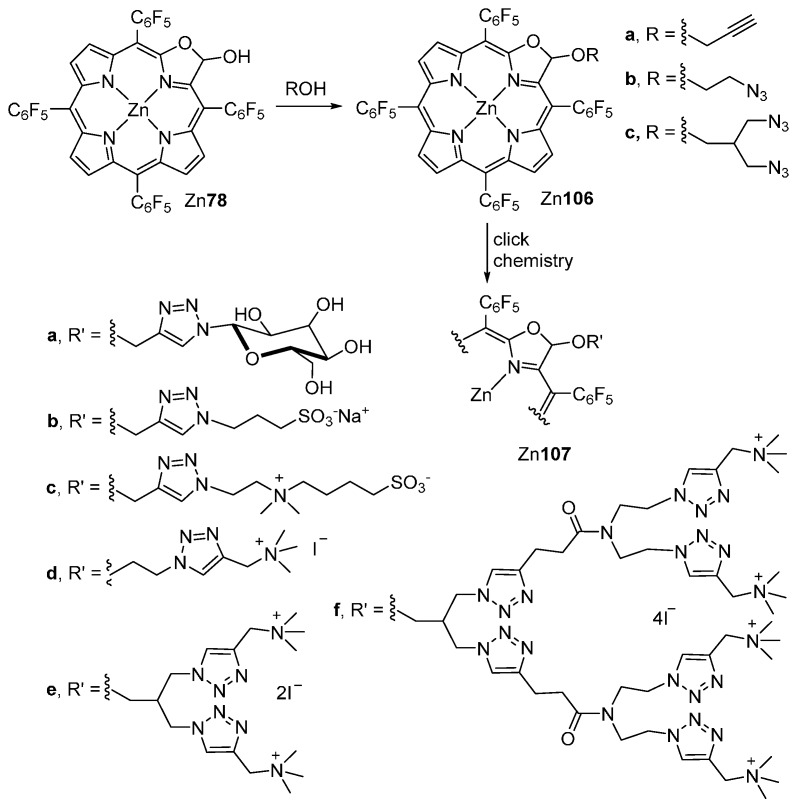
Porpholactol-type compounds studied as photosensitizers in PDT [101].

Recently, Zhang and co-workers [101] reported the synthesis and evaluation of the potentialities of six porpholactol-type compounds (oxazolochlorin acetals) as PDT photosensitizers. The new compounds were prepared by chemical modification of porpholactol Zn**78** (Scheme 38) and bear β-hydrophilic substituents terminated by glucosyl (neutral, Zn**107a**), sulfonic (anionic, Zn**107b**), zwitterionic (Zn**107c**), or ammonium (cationic, Zn**107d**–**f**) groups. It was shown that the terminal ionic groups influence significantly the cellular uptake of these conjugates that is higher for the compounds with positive charge. Importantly, their photocytotoxicity against cancer cells showed IC_50_ values down to the sub-micromolar range being the cationic compounds the most active ones. As revealed by cell imaging experiments, these compounds exert their photodynamic activity through apoptosis.

Porpholactones have also been studied as PDT photosensitizers. Zhang and co-workers compared the photophysical and biological properties of *meso*-tetrakis(pentafluorophenyl)porphyrin (**75**), the corresponding porpholactone **76** and their derivatives bearing thioglucosyl units [102]. This study demonstrated that the β-lactonization of the porphyrin lowers its lipophilicity and increases its binding affinity to low density lipoproteins facilitating its cellular uptake and selective localization within lysosomes. Lactonization also leads to enhanced singlet oxygen quantum yields, thus increasing the intracellular ROS levels. 

The same research group also reported the photosensitizing properties of the two isomeric *cis*- (**77**) and *trans*- (**77’**) porphodilactones (Scheme 27) and the corresponding Zn(II) complexes [89]. The electronic absorption spectra of the two isomers are similar to those of bacteriochlorins, with strong absorption bands in NIR region (>650 nm). However, the authors found that the relative orientation of the two β-lactone moieties has a significant influence on the electronic structures and photophysical properties of the porphodilactones. For example, the Q_y_ band of *trans*-porphodilactone is red-shifted by 19 nm relative to that of the *cis*-isomer, and there is a 2-fold increase in the absorption intensity [89]. The orientation of the two lactone moieties also affects the singlet oxygen production of the two isomeric porphodilactones. The values obtained for the singlet oxygen quantum yields (ΦΔ) were 0.53 for the free-base *trans*-porphodilactone **77’**, 0.66 for Zn**77’**, and 0.87 for Zn**77**. Surprisingly, the free-base *cis*-porphodilactone **77** shows no singlet oxygen production at all after irradiation by a 635 nm laser beam. Both porphodilactones, encapsulated into PLGA nanoparticles, showed no dark toxicity after incubation for 24 h in HeLa cells. However, after irradiation with a 671 nm laser for 60 s, porphodilactones **77’** and Zn**77’** exhibited good phototoxicity with a IC_50_ value of 51 and 44 μg·mL^−1^, respectively. The IC_50_ value of Zn**77** was significantly larger (94 μg·mL^−1^), despite having a larger ΦΔ value, while the free-base *cis*-porphodilactone **77** showed nearly no photocytoxicity toward HeLa cells, which was consistent with the zero singlet oxygen production [89].

### 3.2. Multimodal Imaging Contrast Agents

**Figure 2:**
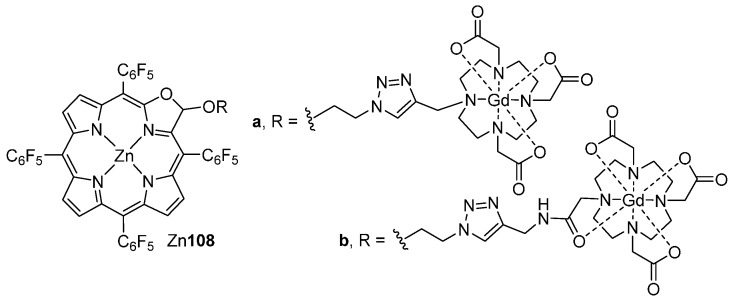
Porpholactol-type compounds studied as multimodal imaging contrast agents [103].

Nowadays, there is a need to develop theranostic compounds for simultaneous cancer imaging, diagnosis and treatment interventions. In this context, and following the procedure indicated in Scheme 38, the group of Zhang prepared two oxazolochlorin acetals bearing gadolinium(III) DOTA-type complexes (Zn**108a**,**b**, Figure 2) [103]. Both compounds can achieve cellular uptake, show no dark cytotoxicity and good photocytotoxicity on HeLa cells. Fluorescence and magnetic resonance imaging experiments showed that both compounds are suitable as bimodal imaging contrast agents. In addition, the photocytotoxicity results indicate that these compounds can also be exploited as photosensitizers for PDT.

### 3.3. Catalysis

The first report about the use of metalloporpholactones in catalysis dates back to 2005, when Cetin and Ziegler [104] demonstrated the catalytic activity of the manganese(III) complex of *meso*-tetraphenylporpholactone, Mn(TPPL)Cl (Mn**5**-Cl), in olefin epoxidation reactions. Comparing with the manganese(III) complex of *meso*-tetraphenylporphyrin, Mn(TPP)Cl, the Mn(TPPL)Cl is a slightly better catalyst for most alkene substrates. However, for the electron-poor substrate hex-1-ene, the turnover number is appreciably larger than the one observed for the manganese porphyrin. 

Iron(III) and manganese(III) complexes of *meso*-tetraphenylporpholactone, Fe(TPPL)Cl and Mn(TPPL)CH_3_CO_2_, are efficient catalysts for the oxidation of sulfides with hydrogen peroxide [105]. Both aliphatic and aromatic sulfides are effectively oxidized to the corresponding sulfoxides and sulfones in yields of 80%–100%. The results show that the iron and manganese porpholactones catalyze the oxidation of sulfides with similar efficiency and are slightly better catalysts than the TPP analogues for most substrates.

The iron(III) complex of *meso*-tetrakis(pentafluorophenyl)porpholactone, Fe(F_20_TPPL)Cl (Fe**76**-Cl), is an effective catalyst for nitrogen atom transfer reactions such as aziridination of alkenes and amidation of alkanes using organic azides [106]. In contrast, the manganese(III) complex of the same porpholactone shows a low catalytic activity.

The palladium(II) complex of *mes*o-tetraphenylporpholactone (Pd**5**) and other Pd(II)-porphyrin complexes were studied as catalysts for the light-induced aerobic oxidation of dibenzylamine to the corresponding imine [107]. The results showed that while PdF_20_TPP could furnish 100% substrate conversion (99% yield) within 1.5 h, the substrate conversion using Pd**5** was only 75% (74% yield) for a similar reaction time. Still, this substrate conversion was higher than the observed for PdTPP (38%, 37% yield), indicating that the lactonization of the porphyrin macrocycle was beneficial for the photocatalysis.

### 3.4. Optical Sensors

Porphyrins and related macrocycles play a preeminent role in sensing applications involving chromophores [108]. Pyrrole-modified porphyrins are also becoming more frequent as chemo(sensors). For instance, the Pt(II) complexes of the *meso*-tetrakis(pentafluorophenyl)porphyrin (Pt**75**) and of the corresponding porpholactone (Pt**76**) have been extensively used as the O_2_-sensitive components of pressure sensitive paints [109,110,111,112,113]. The porphothionolactone **102a** (Scheme 36) is another example. This compound is an effective fluorescent switch-on chemodosimeter for hypochlorite (OCl^−^) [99]. In fact, this porphothionolactone is rapidly and quantitatively converted into the corresponding porpholactone **101a** by the addition of an aqueous solution of NaOCl (bleach). Since the thionolactone possesses only a very dim fluorescence emission but the lactone is brightly fluorescing, this reaction switches the fluorescence on (readily observed with the naked eye) within 10 min at ambient conditions. The conversion of the thionolactone to the lactone is highly selective for the oxidant hypochlorite and that is the basis for the use of this thionolactone as a switch-on chemodosimeter for this anion. However, it must be taken into consideration that this reaction is pH-dependent and acidic to neutral conditions (pH between 1 and 7.4) are required for the conversion of **102a** by OCl^−^ [99].

**Figure 3:**
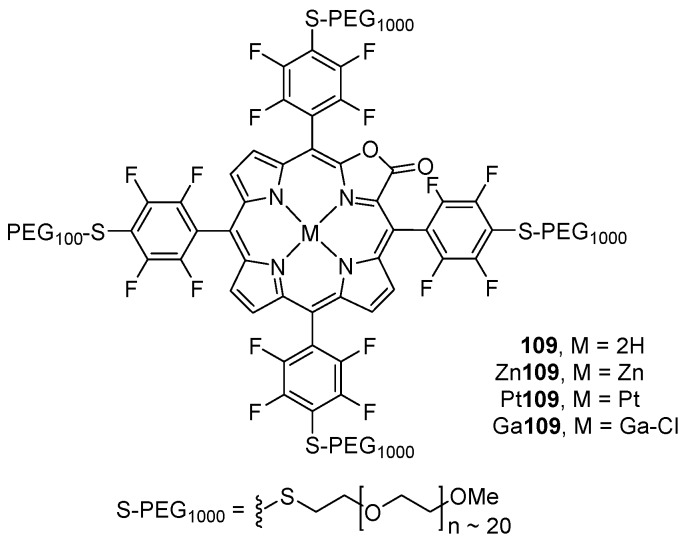
Water-soluble PEGylated porpholactones used as optical cyanide sensors [115].

Cyanide is one of the most toxic and harmful anions to human health and environment but it is still widely used in mining, metallurgy, photographic processing, synthesis of nylon and other synthetic fibers, etc. Thus, it is very important to develop simple, selective, and sensitive methods to the detection of CN^−^, especially in water [114]. One interesting example of the potential applications of the pyrrole-modified porphyrins is their use as optical cyanide chemosensors. Recently, the group of Brückner demonstrated that the water-soluble PEGylated porpholactone **109** and its zinc(II), platinum(II), and gallium(III) complexes (Figure 3) behave as optical cyanide chemosensors in aqueous solutions, albeit these sensors possess only modest sensitivities [115]. It was shown that the optical response of the Zn**109** complex to cyanide is totally different from that of the free-base, platinum, and gallium complexes. While for the Zn**109** it can be attributed to axial ligation of the cyanide to the central metal, the sensing mechanism for **109**, Pt**109** and Ga**109** relies on a nucleophilic addition of the cyanide to the lactone moiety. Incorporation of the gallium porpholactone complex (Ga**109**) in a Nafion® membrane resulted in a material that shows a reversible response to CN^−^ and remains stable and transparent even after days in aqueous solutions [115].

**Figure 4:**
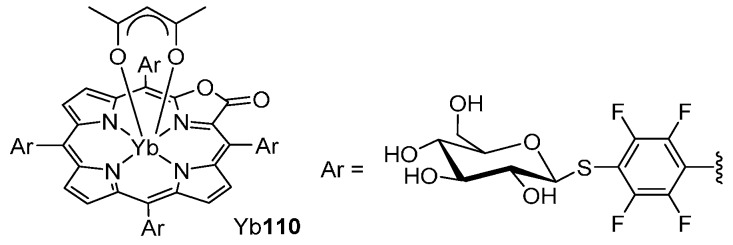
Water-soluble NIR luminescent Yb(porpholactone) complex [116].

A recent study demonstrated that β-lactonization of porphyrin ligands enhances the sensitization efficiency of the NIR luminescence of the ytterbium(III) complexes (Figure 4) [116]. In addition, it was found that the NIR emission of the water-soluble Yb(porpholactone)-glucose conjugate Yb**107** is specifically switched on in the presence of glucose oxidase and is switched off by addition of glucose. This experiment shows that ytterbium complex Yb**110** may find application as a switchable probe emitting in the NIR region in which biological tissues and fluids are relatively transparent (900–1100 nm).

The ability of *meso*-tetrakis(pentafluorophenyl)porpholactone (**76**) and its Pt(II) complex to function as optical high pH sensors was reported by the group of Brückner [53]. This group found that these compounds behave as chemosensors for OH^−^ and MeO^−^. When submitted to strong alkaline or high methoxide conditions their UV-vis spectra undergo dramatic and reversible red-shifts. The dynamic range for the Pt**76** in solution is from pH 11.5 to 13.2. This complex can also be used to coat optical fibers, allowing the construction of optical fiber-based pH sensors.

This study was extended to a range of metal complexes of other *meso*-tetraarylporpholactones, allowing to investigate the influence of the metal (M = Zn(II), Ni(II), Cu(II), Pd(II), Ag(II), Pt(II)), the aryl group (Ar = Ph, C_6_F_5_), and the presence of a β-NO_2_ group, on the pH sensing range [117]. The results showed that the optical response for the pentafluorophenyl-substituted derivatives is stronger than that of the nitrated phenyl-substituted analogues. However, β-nitration and pentafluorophenyl-substitution have additive effects. Since the metal has a minor influence on the optical response, the cheaper and easier to prepare zinc, copper and silver complexes of porpholactones are excellent alternatives to the platinum complexes [117].

PEGylated and fully water-soluble Pt(II)- and Ga(III)porpholactones behave as high pH sensors in purely aqueous solutions (pH between 10 and 12.5 and 8 and 11, respectively) [118]. Incorporation of these compounds into a Nafion^®^ optode membrane resulted in sensors suitable for a moderately rapid (response time in minutes) sensing of high concentrations of hydroxides (pH 11 and above).

## 4. Conclusions 

As discussed above, the pyrrole-modified porphyrin chemistry is evolving quickly. Several methods to access these compounds, and new reactions for their conversion into other porphyrinoids, were already reported. Also, various (potential) applications have been suggested for these types of compounds and, in many cases, these porphyrinoids show a better performance than their porphyrin analogues. However, considering that these studies have been developed by a very restricted number of research groups, for a faster development of the pyrrole-modified porphyrins field (chemistry and applications) it is crucial that other research groups may become interested in this topic. Thus, we expect that this review contributes to motivate other researchers to embrace this subject.

## Figures and Tables

**Table 1:** Yields of the oxidation of 2,3-dioxochlorins M**44** under different conditions ^1^.

M44	BSA	Reaction Time	M46	M5	M44 Recovered
Cu**44**	2 equiv	22 h	8%	27%	59%
Cu**44**	4 equiv	8 h	7%	70%	11%
Cu**44**	4 equiv	18 h	traces	75%	—
Cu**44**	8 equiv	5 h	6%	82%	traces
Ni**44**	4 equiv	14 h	18%	14%	65%
Ni**44**	6 equiv	9 h	19%	35%	20%

^1^ Data from ref. [69].

## Abbreviations

BSA: benzeneselenic anhydride
CTAP: cetyltrimethylammonium permanganate
DDQ: 2,3-dichloro-5,6-dicyano-1,4-benzoquinone
DOTA: 1,4,7,10-tetraazacyclododecane-1,4,7,10-tetraacetic acid
PDT: photodynamic therapy
Pic: 2-picolinic acid
PLGA: poly(lactic-co-glycolic acid)
ROS: reactive oxygen species
TPP: *meso*-tetraphenylporphyrin
H_2_F_20_TPP: *meso*-tetrakis(pentafluorophenyl)porphyrin
*p*-TSA: *p*-toluenesulfonic acid
